# Heart Rate Variability Biofeedback Intervention Programme to Improve Attention in Primary Schools

**DOI:** 10.1007/s10484-024-09659-w

**Published:** 2024-08-23

**Authors:** Ainara Aranberri Ruiz, Borja Nevado, Malen Migueles Seco, Aitor Aritzeta Galán

**Affiliations:** Department of Basic Psychological Processes and their Development, Faculty of Psychology, University of the Basque Country UPV/EHU, Donostia-San Sebastián, Spain

**Keywords:** Attention, Intervention, Primary school, Biofeedback, Heart rate variability, Breathing

## Abstract

The importance of attentional capacity for academic performance is highlighted by the increasing demands placed on students during primary school. Between the ages of 7 and 12, there is an evolutionary improvement in attentional capacity and the school environment is considered an appropriate setting in which to develop programmes to improve attention. Heart rate variability is an appropriate indicator of attentional capacity. For all these reasons, a heart rate variability biofeedback intervention focused on breathing was developed and implemented to improve attention. The intervention consisted of two phases. In the first phase, the school teachers were trained to develop the intervention; in the second, students received five individual sessions from their teachers. In each individual session, they learned to breathe to increase their heart rate variability. A total of 272 girls and 314 boys (N = 586) aged 7–12 years participated in the programme. To study the impact of the intervention on three primary school age groups, the attention of Control and Experimental groups was assessed before and after the implementation of the programme. According to the data obtained, despite developmental improvements, the students who participated in the programme showed an increase in heart rate variability and an improvement in attentional capacity, with a greater impact on the first cycle of primary school. The usefulness of heart rate variability biofeedback interventions in improving attention in primary school is discussed and arguments for their use in children are presented.

## Introduction

During the school years, a child’s attentional demands increase and expand to include more symbolic stimuli (Ristic & Enns, 2015; Santa-Cruz & Rosas, 2017). Specifically, in the primary school cycle, new school demands require students to perform higher cognitive tasks (Mikhailova, 2017; Schachner, 2019), such as reading and mathematical problem-solving (Kim et al., 2018). Such tasks are strongly related to attentional capacity, which is crucial for learning (Duarte et al., 2020; Fisher et al., 2013; Rabiner et al., 2016).

Attention is the activity of three brain networks (vigilance, orientation, and executive control) that influence how information is processed (Posner, 2012; Posner et al., 2020). The *vigilance network* is responsible for the state of alertness, enabling faster reaction times once action is needed. The *orientation network* focuses on locating specific sources of stimulation and allows the efficient and rapid selection of the correct modality and location for primary sensory processing. Finally, the *executive control network* is the system that provides focal attention, the limited awareness of relevant information that inhibits the processing of other input and enables the complex neurostructural activation system that constitutes consciousness (Blaser et al., 2023; Peterson & Posner, 2012). All three attentional networks interact with each other, and in different ways, to influence attentional performance (Spagna et al., 2014; Xuan et al., 2016) and are necessary for proper academic performance (Posner, 2023; Posner et al., 2020). Such networks are present in the infant brain but at a lower degree of functional integration than in the adult brain (De Bie et al., 2012; Kaufmann et al., 2017; Posner, 2023). Integrations of these processes increase to a greater degree during infant development (De Bie et al., 2012), enabling better attentional performance (Posner et al., 2020; Rohr et al., 2018).

The d2 Test of Attention (Brickenkamp & Zillmer, 1998) is one of the most widely used neuropsychological tests for measuring attention (Arán Filipetti et al., 2022); it provides measures of selective attention, inhibitory control, and concentration (Brickenkamp, 2002). These cognitive skills are associated with the aforementioned attention network of Posner's model (Blair & Ursache, 2011; Posner & Rothbart, 2007; Rueda et al., 2012) and, specifically, the orienting network, which encompasses the selective attention and inhibitory control (Petersen & Posner, 2012) necessary for D2 performance. A normative study by Jiménez et al. (2012) observed that, in primary school children aged 6–12 years, there was an improvement in attentional ability—measured by the d2 test of attention (Brickenkamp, 2002)—as development progresses, which was supported by another recent study (Arán Filipetti et al., 2022).

Given that attention is under a developmental process during this period (Arán Filipetti et al., 2022; Jiménez et al., 2012; Pozuelos et al., 2019; Rivera et al., 2017), the elaboration of interventions aimed at improving attentional skills may be particularly appropriate given their potential to improve school programmes (Karbach & Unger, 2014a, 2014b; Lee et al., 2019; Zhang & Bray, 2020) and their capacity to provide opportunities for curricular improvement (Rueda et al., 2012).

Heart rate variability (hereafter HRV) refers to changes in the time interval that occur between consecutive heartbeats (Shaffer & Ginsberg, 2017; Shaffer et al., 2023; Task Force of the European Society of Cardiology, 2016) and is related to the functioning of the autonomic nervous system (Aranberri-Ruiz, 2023). Vagus nerve-mediated HRV is an indicator of parasympathetic activity (Blaser et al., 2023; Laborde et al., 2017; Thayer & Lane, 2000). Respiration directly affects HRV (Vaschillo et al., 2006), the phenomenon where heart rate increases with inhalation and decreases with exhalation (Berntson et al., 1993; Eckberg, 1983). HRV can be maximized through slow-paced breathing (Laborde et al., 2017). In this way, HRV is an appropriate indicator of the stress response (Aranberri, 2023; Aranberri et al., 2022; Aritzeta et al., 2022; Aritzeta et al., 2017; Pine & Bruckner et al., 2023); of the level of cognitive function (Thayer et al., 2009; Winkelmann et al., 2017); and is also considered a measure of brain regulatory capacity (Kumral et al., 2019; Mather & Thayer, 2018; Schuman et al., 2021). Furthermore, HRV has been proven to be an appropriate measure of attention (Forte et al., 2019; Jennings et al., 2016; Ning & Wang, 2021; Park & Thayer, 2014; Porges & Raskins, 1969; Sakaki et al., 2016; Thayer & Lane, 2009; Tinello et al., 2022). Practising slow-paced breathing, e.g., with HRV biofeedback interventions, over a longer period of time (e.g., four weeks) has been shown to have a wide range of positive emotional and cognitive effects (Goessl et al., 2017; Lehrer et al., 2020). Through biofeedback techniques, we obtain real-time information about variations in HRV (Schwartz & Andrasik, 2003) and we can learn to modulate our HRV by practising slow and prolonged breathing (Goessl et al., 2017). The Polyvagal Theory (Porges, 1995, 2011, 2022) and Thayer and Lane (2000) in their Model of Neurovisceral Integration justify the impact of slow and prolonged breathing on the ventral vagus nerve and its parasympathetic influence, reducing the heart rate and increasing HRV itself by reducing the activity of the adrenal sympathetic system and the consequent stress response (Aranberri-Ruiz, 2023), thus making it possible to improve attentional capacity (Kredlow, et al., 2022).

Specifically, HRV biofeedback programmes focus on learning a breathing pattern of approximately six breaths per minute—a measure also validated through studies of the impact of the breathing pattern on evoked action potentials of different brain areas (Herrero et al., 2017)—have been proven effective in improving academic-cognitive performance and attentional capacity (Aritzeta et al., 2017; Park et al., 2013; Rush et al., 2017). To our knowledge, only two HRV biofeedback interventions are known to improve attention in Primary Education (Crevenna et al., 2016; Rush et al., 2017). Crevena et al. (2016) recruited 15 pupils in the fourth year of Primary School (10 years old), whereas the intervention by Rush et al. (2017) was aimed at 27 pupils aged 8 to 12 years. In both studies, students improved their attentional capacity after the training. However, the sample sizes of these studies were very small and did not allow the evaluation of the differential effectiveness of the treatment in the three cycles comprising Primary Education in a single study.

Thus, given the effectiveness and scarcity of HRV biofeedback interventions in school settings, an HRV biofeedback programme focused on breathing was designed to improve the attentional capacity of primary school students. We expected, as in previous studies and independently of the educational cycle, that the training would improve performance according to the d2 test of attention (Brickenkamp, 2002). The study further aimed to examine the interactions of training with educational level in different attention measures.

## Material and Methods

This study included 585 primary school students (46.4% girls; 53.5% boys) aged between 7 and 12 years (M = 8.51; SD = 1.26). In primary education, each cycle consists of two courses: the first cycle consists of first and second courses, the second cycle of third and fourth courses, and the third cycle of fourth and fifth courses. The sample was divided according to the cycles of primary schooling into three age groups, with 21.4% in the first cycle (Age Group 1; ages 7–8), 64.6% in the second cycle (Age Group 2; ages 9–10), and the remaining 14% in the third cycle (Age Group 3; ages 11–12).

To carry out the study, the sample was divided into Experimental and Control groups. Regarding the composition of the Experimental group, at the suggestion of school management, it was decided to assign students with different difficulties (emotional, academic, etc.) to the Experimental group. The selection process involved tutors, teaching staff, the head of therapeutic education, and the management team. The rest of the students were randomly assigned. Thus, in Age Group 1, there were 83 participants in the Experimental group and 42 in the Control group, Age Group 2, had 257 participants in the Experimental group and 121 in the Control group, and, finally, Age Group 3 included 49 participants in the Experimental group and 33 in the Control group.

The participation of the students was voluntary and consented to by the school council, parents, and guardians. The study had the favourable report of the ethics committee for research with human beings, their samples, and data (CEISH/269 1–2-3–4-/2014) of the University of the Basque Country/Euskal Herriko Unibertsitatea; DSI file INA0079. The ethical aspects required for research with human subjects (informed consent, right to information, protection of personal data, guarantees of confidentiality, non-discrimination, free of charge, and the possibility of abandoning the study at any stage) were scrupulously respected.

### Design

The biofeedback treatment to teach girls and boys to take prolonged and paused breaths (approximately six breaths per minute) across five individual sessions: the first measure (baseline) allowed us to establish differences before treatment, and the last measure was the final treatment or post-treatment measure (see Table 1).

**Table 1:** Intervention design

Groups	Week 1	Week 2	Week 3	Week 4	Week 5
Experimental	CS and d2 (pre-test) Training	Training	Training	Training	Training CS and d2 (post-test)
Control	CS and d2 (pre-test) No training	No training	No training	No training	No training CS and d2 (post-test)

*CS* coherence score

### Procedure and Instruments

HeartMath EmWave software (Institute of HeartMath, 2012) was chosen in this study to evaluate the effects of an HRV biofeedback programme on attention tasks and to teach prolonged and paused breathing. This software was proven effective in several studies (Aranberri et al., 2022; Aritzeta et al., 2022; Aritzeta et al., 2017; Rush, et al., 2017; Idris et al., 2017; Pine & Bruckner, 2023), measuring HRV in real-time with a sensor placed on the participant's earlobe. Thus, the computer, through on-screen images, offers HRV values in real-time, thus allowing the subject to observe the impact that the breathing pattern itself has on HRV. Using different software applications, the children learnt, through trial, error, and success, to breathe in a prolonged and paused manner (approximately six breaths per minute), thus increasing their own HRV. Based on HeartMath EmWave software (Institute of HeartMath, 2012), an HRV biofeedback programme for attentional improvement was developed in two implementation phases.

To analyse the effects of the programme, as in the aforementioned studies (Aranberri et al., 2022; Aritzeta et al., 2022; Aritzeta et al., 2017; Idris et al., 2017; Pine & Bruckner, 2023), we used the Coherence Score (CS) provided by the EmWave programme. Coherence refers to a physiological state involving a balance between the parasympathetic and sympathetic nervous systems, with a possible relative increase in parasympathetic activity. It is typically indicated by a "large, characteristic spectral peak" recorded at around 0.1 Hz in the low-frequency band. A CS is a ratio based on a proprietary algorithm that reflects the level of coherence calculated at five-second intervals (HeartMath, n.d.). HeartMath divides coherence into three levels (low, medium, and high) based on parasympathetic responding, with low corresponding to a strong presence of stress and high to a lack-of-stress state. A coherence ratio score represents the proportion of total session time spent at each level (HeartMath, Inc., 2020). A high-level score represents high HRV states, which are associated with relaxed states, i.e., the state we want to achieve through deep breathing.

Phase 1 or pre-intervention. Consisted of theoretical and practical training with the aforementioned computer application—HeartMath EmWave software (Institute of HeartMath, 2012)—for the school’s teaching staff, thus providing the necessary training for teachers to be able to carry out the training programme developed for each individual pupil, described below in Phase 2.

Phase 2 or intervention programme. This consisted of six weekly sessions: the first was performed in-group and the remaining five individually. In the first session in each classroom participating in the programme, the tutor, with a member of the research team, explained the intervention to all the students in a pleasant way. After one week, individual training in HRV biofeedback began for each student. The five individual sessions were carried out with the tutor of each student in a relaxed and suitable place to develop the intervention. There were two chairs, one for the tutor and one for the student, a table with a computer in which the HeartMath EmWave software (Institute of HeartMath, 2012) was installed, and each computer had its corresponding earlobe sensor to detect HRV. Each session lasted for 20 min, and after each session, the tutors recorded the HRV values obtained by each participant on each student's record sheet. During the five sessions, and using the Coherence Coach and Balloon Game applications—which resemble the animations and cartoons of the HeartMath EmWave software (Institute of HeartMath, 2012)—the children learnt to breathe deeply and slowly (approximately six pairs of breaths per minute) by trial, error, and success, performing different actions from session to session. In each session, the student must learn that in any place where they feel nervous (school, home, street…), breathing deeply and slowly will make them feel better. So, at the beginning and end of each session, the teacher explained: “*We are going to breathe in a relaxed way to feel better. In whatever situation you feel bad, you have to breathe deeply and everything will be better*”. To learn to breathe deeply and slowly, the student only worked with the emWave programme in the first and second sessions. In the third session, to generalise what had been learnt, each student was given a 'target' image—specific to the programme—laminated in a 6 × 4 cm format so that they could carry it with them in their school bag and use it when the teachers recommend it and when they felt nervous. In this way, the image helped them to breathe deeply, slowly, and for a long time, without the need for the computer programme. In the remaining sessions, 4 and 5, they continued to practise the breathing they had learnt using the target picture. In the last session, session 5, the intervention ended by congratulating each pupil who had taken part, stressing the importance of the breathing exercises they had learnt to feel better, and encouraging them to use the image wherever they are (street, home, school…), emphasising that once they have learnt to breathe deeply, it is not necessary to carry the image with them.

To assess the attentional capacity before and after training, both the Control and Experimental groups applied the d2 test of attention (Brickenkamp, 2002). This test measures attention and was designed for people aged between 6 and 60 years. It is composed of 14 lines, each with 47 characters, for a total of 658 items. Participants must identify any letter "d" that has two dashes (one at the top and bottom, both at the top, and both at the bottom). These are the relevant items, while the other combinations (the 'p' with or without dashes and the ‘d’ with or without dashes) are irrelevant. The participants have 20 s per line, and it is the instructor who tells them when to start and finish. The dimensions considered in this study were: Total correctly processed (TN-E); omissions (O)—total number of relevant items not marked—as well as the TOTR count, which is calculated by subtracting the sum of omissions (O) and errors (E)—TN-E and measures the total effectiveness of the test; and concentration (CON), which is calculated by subtracting errors (E) from TN-E. The psychometric properties of the d2 test were suitable (average reliability coefficient of 0.95).

### Analysis and Results

First, normality was assessed using the Kolmogorov–Smirnov test, showing that data was distributed normally. Results were analysed with 2 × 2x3 ANOVAS for mixed designs, 2 (Group: Control, Experimental), × 2 (Assessment: pre- and post-training), × 3 (Age Group: 1, 2 and 3) with the variables Group and Age Group as independent measures, and Assessment as a repeated measure. *Post-hoc* comparisons were performed with the Bonferroni test and pairwise comparisons with the Student's t-test.

#### Coherence Score (CS)

CS measures (low, medium, and high) were analysed separately to evaluate the differential impact of intervention on the parasympathetic system.

#### Low CS

ANOVA analysis revealed a significant difference between pre- and post-scores (main effect Assessment), *F*(1, 552) = 92.86, *p* = 0.000, ${\eta }_{p}^{2}$ = 0.144, where low CS was reduced over time (Pre: 63.86 vs. Post: 48.2). Furthermore, treatment did create differences among groups (Main effect group), *F*(1, 552) = 27.686, *p* = 0.000, ${\eta }_{p}^{2}$ = 0.048, as scores in the Experimental group were lower than in the Control group (Experimental: 49.17 vs. Control: 62.89). There were some differences among all three age groups (Age Group 1: 58.5 vs. Age Group 2: 52.722 vs. Age Group 3: 56.87) however said differences were not reliable (Main effect Age Group), *F*(2, 552) = 2.845, *p* = 0.059, ${\eta }_{p}^{2}$ = 0.010.

**Fig. 1:**
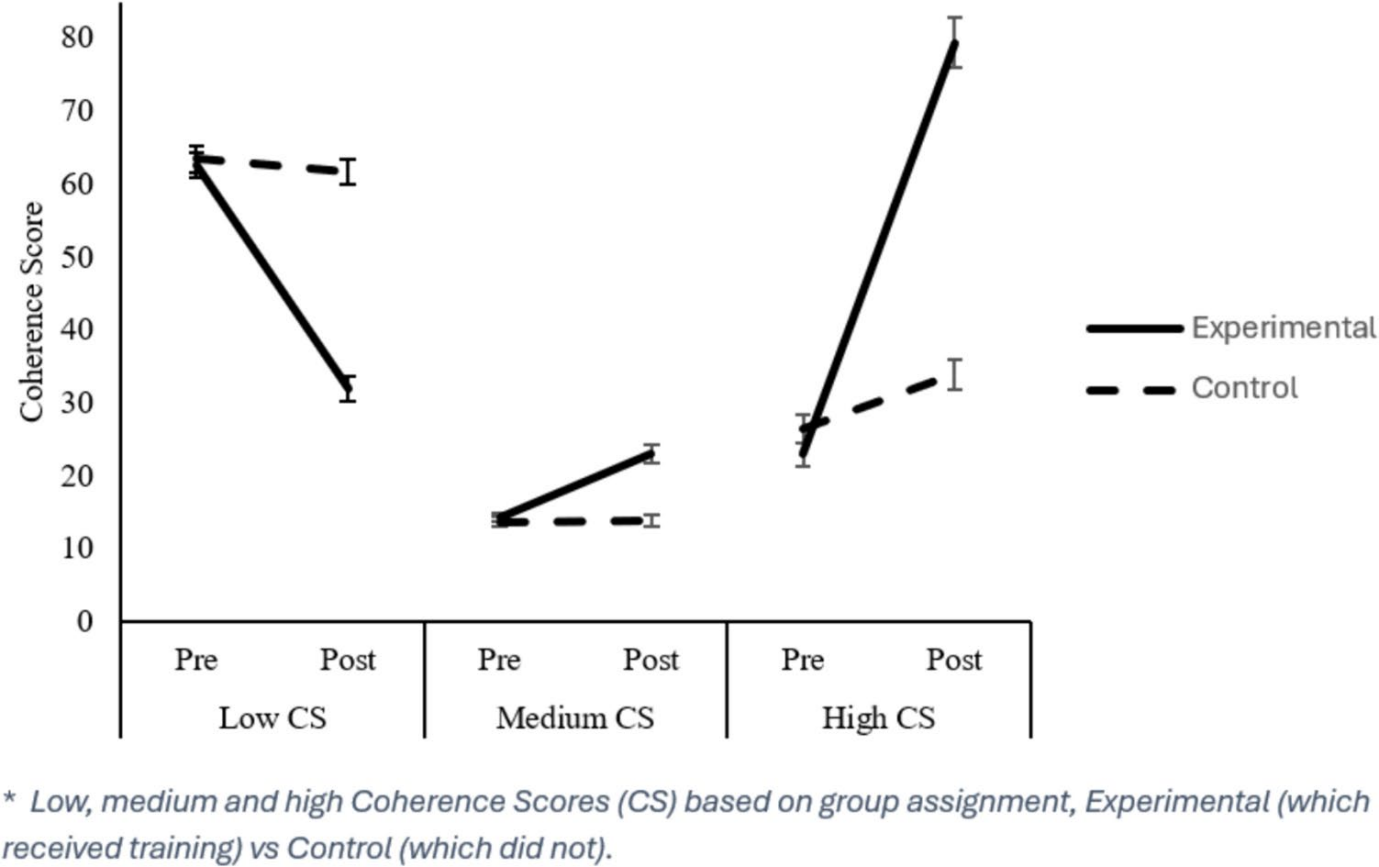
Difference from Pre to Post for all the CS variables based on group assignment

Regarding interactions, pre- to post-treatment scores varied based on Group (Assessment x Group interaction; see Fig. 1, left), *F*(1, 552) = 80.83, *p* = 0.000, ${\eta }_{p}^{2}$ = 0.128, changes from pre- to post-treatment in the Experimental group (Pre: 64.31, Post: 34.03) were significantly higher than those in the Control group (Pre: 63.41, Post: 62.36). Moreover, variation from pre- to post-treatment did vary based on Age Group (Assessment x Age Group interaction), *F*(2, 552) = 5.68, *p* = 0.004, ${\eta }_{p}^{2}$ = 0.020, and both Age Group and Group (Assessment x Age Group x Group), *F*(2, 552) = 4.9, *p* = 0.008, ${\eta }_{p}^{2}$ = 0.017.

Thus, before intervention, Age Groups did not differ (*p’s* ≥ 0.785); however, afterwards, participants in Age Group 1 (M = 52. 23) benefited significantly less than those in Age Group 2 (M = 42.94; *p* = 0.002) but not compared with those in Age Group 3 (M = 46.59; *p* = 0.473). There were no differences between Age Groups 2 and 3 (*p* = 0.881). Moreover, groups did not differ in any Age Group before intervention (*p* ≥ 0.337); however, differences manifested afterwards in all three groups, t ≤ -3.27, *p* ≤ 0.001, d ≤ -0.56, with Age Group 2 presenting the greatest improvement (Experimental: 25.17 vs. Control: 60.63), followed by Age Group 3 (Experimental: 30.95 vs. Control: 63.57), then Age Group 1, in which the intervention was the least beneficial (Experimental: 45.83 vs. Control: 62.92), yet beneficial nonetheless.

#### Medium CS

Analysis of the medium CS measure ANOVA revealed the main Assessment effects, *F*(1, 552) = 23.65, *p* = 0.000, ${\eta }_{p}^{2}$ = 0.41, Group, *F*(1, 552) = 16.107, *p* = 0.000, ${\eta }_{p}^{2}$ = 0.028, and Age Group, *F*(2, 552) = 7.53, *p* = 0.001, ${\eta }_{p}^{2}$ = 0.027. Thus, in general, there was in increase CS across phases (Pre: 14.06 vs. Post: 18.98), among groups (Experimental: 18.65 vs. Control: 14.38) and age groups (Age Group 1: 13.66 vs. Age Group 2: 16.97 vs. Age Group 3: 18.92).

Interaction analysis yielded a significant Assessment x Group interaction (see Fig. 1, centre), *F*(1, 552) = 9.04, *p* = 0.003, ${\eta }_{p}^{2}$ = 0.016. The Experimental group experienced a significant increase in medium HRV after treatment (Pre: 14.67, Post: 22.63) compared with the Control group (Pre: 13.45, Post: 15.32), which experienced no such increase. No other interactions were found, *F* ≤ 2.905, *p* ≥ 0.056.

#### High CS

The prior analysis applied to the High CS score revealed the main effects of Group, *F*(1, 554) = 20.77, *p* < 0.000, ${\eta }_{p}^{2}$ = 0.036, and Assessment, *F*(1, 554) = 146, *p* = 0.001, ${\eta }_{p}^{2}$ = 0.209, yet no effect of Age Group, *F*(1, 554) = 1.90, *p* = 0.15. Regarding interactions, Assessment did interact with group assignment (Assessment x Group interaction; see Fig. 1, right), *F*(1, 554) = 106.99, *p* = 0.001, ${\eta }_{p}^{2}$ = 0.162, as well as Age Group (Assessment x Age Group interaction), *F*(2, 554) = 19.72, *p* = 0.001, ${\eta }_{p}^{2}$ = 0.066, and there was a three-way interaction (Assessment x Group x Age Group interaction), *F*(2, 554) = 9.05, *p* = 0.001, ${\eta }_{p}^{2}$ = 0.032.

Thus, despite Age Groups not differing in general, as manifested by the lack of a main Age Group effect, they did benefit differentially from the intervention, as manifested by the three-way interaction. Experimental Age Group 1 had a lower pre-treatment performance, t(140) = −2.12, *p* = 0.036, d = −0.37, compared with the Control group (Experimental: 26.61 vs. Control: 37.4), however, after intervention, their performance was similar (Experimental: 49.77 vs. Control: 38.4), t(140) = 1.76, *p* = 0.081. Age Group 2, however, did not show any differences pre-intervention, t(346), *p* = 0.646, which became apparent afterwards (Experimental: 94.56 vs. Control: 33.18), t(342) = 11.55, *p* = 0.000, d = 1.25. Lastly, Experimental Age Group 3 had a poorer performance in the pre-test measure compared with the Control Group (Experimental: 13.13 vs. Control: 31. 38), t(74) = −2.97, *p* = 0.005, d = −0.68. Nevertheless, post-intervention, the former not only improved but actually outperformed their counterparts (Experimental: 76.39 vs. Control: 31.71), t(73) = 4.30, *p* = 0.000, d = 0.99.

Performance in the attention test was assessed by scoring one point for each mark made, whether correct or incorrect. In addition, omissions or unmarked stimuli were also scored. Thus, to examine the impact of HRV biofeedback training on the attentional performance of girls and boys in the d2 test, we analysed the hit rate, omissions, task concentration, and total test effectiveness (see Table 2).

**Table 2:** Means and (standard deviations) of the Experimental and Control groups in the pre- and post-tests for the three primary education Age Groups

		Experimental			Control		
		Age group 1	Age group 2	Age group 3	Age group 1	Age group 2	Age group 3
		*M(DT)*	*M(DT)*	*M(DT)*	*M(DT)*	*M(DT)*	*M(DT)*
*TN-E*	Pre	57.13 (26.13)	96.76(41.13)	136.79(44.96)	73(27.00)	103.14(32.16)	153.85(33.73)
	Post	85.32(27.06)	120.02(38.39)	165.68(42.41)	68.87(30.29)	118.92(41.23)	154.35(54.39)
*CON*	Pre	43.23(28.50)	89.56(48.20)	132.75(47.36)	42.78(37.28)	95.92(35.04)	148.36(45.41)
	Post	76.57(30.28)	116.04(39.87)	163.51(44.75)	51.72(50.75)	121.09(37.07)	163.67(37.75)
*TO*	Pre	243.11(27.89)	201.90(41.49)	162.21(44.95)	225.22(26.66)	195.86(31.94)	145.09(33.82)
	Post	213.22(27.46)	167.40(56.43)	127.86(49.23)	229.97(30.31)	152.42(69.10)	116(55.81)
*TOTR*	Pre	57.13(26.13)	96.76(41.13)	136.79(44.96)	73(27.00)	103.14(32.16)	153.85(33.73)
	Post	85.32(27.06)	120.55(37.64)	165.68(42.41)	68.87(30.29)	123.59(34.42)	165.78(34.89)

total correctly processed* (TN-E)*, *CON* concentration (total hits-errors), *TO* total omissions, *TOTR* total responses—(omissions + errors)

#### Total Correctly Processed (TN-E)

The TN-E measure refers to the number of relevant characters marked correctly. The number of correct answers significantly changed after manipulation (Main effect Assessment), *F*(1, 490) = 89.08, *p* = 0.001, ${\eta }_{p}^{2}$ = 0.154, as correct answers increased in the last evaluation (Pre: 98.27 vs. Post: 119.17). Differences among Age Groups were also significant (Main effect Age Group), *F*(1, 490) = 120.49, *p* = 0.001; however, the group factor was not, *F*(1, 490) = 0.176, *p* = 0.675. Despite the non-significant group effect, there was an interaction between group and timing of evaluation (Assessment x Group effect), *F*(1, 490) = 45.53, *p* = 0.001, ${\eta }_{p}^{2}$ = 0.085. Thus, although in the initial evaluation, the Control group obtained more correct scores in the attention test than the Experimental group (Control: 105.48 vs. Experimental: 94.01), *t*(552) = −2.93, *p* = 0.004, d = 0.268), after training, there were no significant differences between the Control and Experimental groups (Control: 113.61 vs. Experimental: 118.62), *t*(523) = 1.19, *p* = 0.236, d = 0.18. However, each group improved in the total number of correct scores from the initial to the final assessment, showing a greater impact of training on the Experimental group, *t*(330) = −15.81, *p* = 0.000, d = −0.580, compared with the Control group*, t*(164) = −3.58, p = 0.000, d =−0.220). In addition, the Age Group factor was significant (Main effect Age Group), *F*(2,490) = 120, *p* = 0.000, ${\eta }_{p}^{2}$ = 0.330, *Post-hoc* comparisons with the Bonferroni test showed that as the age of the students increased, performance regarding the number of correct answers increased in the attention test. Thus, students in Age Group 1 performed worse (M = 71.31) than students in Age Groups 2 (M = 111.40) and 3 (M = 152.81). Moreover, students in Age Group 3 also outperformed students in Age Group 2 (*p* = 0.000). Finally, the three-way interaction Group x Age Group x Assessment was significant, *F*(2,490) = 10.33, p = 0.000, ${\eta }_{p}^{2}$ = 0.040. An important aspect to highlight is the particularly positive impact shown by the intervention on Age Group 1 concerning the improvement in the total number of correct scores obtained. On one hand, despite the Experimental group obtaining worse scores in total hits than the Control group in the pre-test, it should be noted that Age Group 1 was the only one (all other Age Groups, *p’s* ≥ 0. 313) in which the Experimental group showed an improvement in post-test scores compared with the Control group (Experimental 85.32 vs. Control: 68.87),* t*(113) = 2.86, *p* = 0.006, d = 0.574. Moreover, in Age Group 1, the Experimental group obtained a statistically significant improvement from the first assessment to the last assessment, with a large effect size, *t*(66) = −8.135, p = 0.000, d = −1.204, higher than the other Age Groups, which obtained moderate effect sizes, Age Group 2: *t*(216) = -11.82, *p* = 0.000, d = −0.610; Age Group 3: *t*(44) = −6.95, *p* = 0.000, d = −0.689.

#### Omissions (TO)

The TO measure refers to the total number of relevant items not checked. The number of Omissions was lower in the post-test than in the pre-test (Post: 165.949 vs. Pre: 201.002), *F*(1,521) = 99.89, *p* = 0.001, ${\eta }_{p}^{2}$ = 0.161. The Group x Assessment interaction was also significant *F*(1,521) = 5.43, *p* = 0.002, ${\eta }_{p}^{2}$ = 0.020. Thus, in the initial evaluation, the Control group committed fewer omissions than the Experimental group (Control: 193.94 vs. Experimental: 205.02), *t*(552) = 2.96, *p* = 0.003, d = 0.272, while in the final evaluation, there were no statistically significant differences between Control and Experimental groups (Control: 162.51 vs. Experimental: 171.61), *t*(553) = 1.64, *p* = 0.101. Both the Control and Experimental groups improved their performance from the initial to the final assessment, however, the improvement was more prominent in the Experimental group, Control: *t*(179) = 6.98, *p* = 0.000, d = 0.606; Experimental: *t*(346) = 13.28, *p* = 0.000, d = 0.775). The Age Group factor was significant, *F*(2,521) = 123.91, *p* = 0.000, ${\eta }_{p}^{2}$= 0.322. *Post-hoc* comparisons with the Bonferroni test showed that as the age of the students increased, performance in the TO dimension improved with the number of omissions decreasing. Thus, students in Age Group 1 performed worse (M = 227.586) than students in Age Group 2 (M = 180.1664) and Age Group 3 (M = 139.203). Moreover, students in Age Group 3 also outperformed students in Age Group 2 (all *p’s* = 0.000). We observed significant interactions between Assessment time x Age Group, *F*(2,521) = 9.82, *p* = 0.000, ${\eta }_{p}^{2}$=0.036, and between Assessment time x Group x Age Group, *F*(2,521) = 7.94, *p* = 0.000, ${\eta }_{p}^{2}$ = 0.030. Recalling that the Experimental group obtained worse pre-test scores than the Control group, in the post-test, the Age Group 1 Experimental group obtained better scores (Experimental: 213.22 vs. Control: 229.97), *t*(113) = -2.89, *p* = 0.005, d = -0.580). Moreover, the Age Group 1 Experimental group obtained better scores in the post-test than in the pre-test with a large effect size, *t*(66) = 7.97, *p* = 0.000, d = -1,230, higher than in the other Age Groups: Age Group 2: *t*(230) = 9,916, *p* = 0.000, d = 0.746; Age Group 3: *t*(46) = 6.49, *p* = 0.000; d = 0.753.

#### Concentration (CON)

The CON parameter is a measure of accuracy calculated by subtracting errors from the total number of hits. Analyses showed that there was a higher Concentration in the final evaluation than in the initial evaluation (115.646 vs. 89.886), *F*(1,485) = 162.090, *p* = 0.000, ${\eta }_{p}^{2}$ = 0.250. The Group factor was not significant but interacted with the Evaluation factor, *F*(1,485) = 10.04, *p* = 0.000, ${\eta }_{p}^{2}$ = 0.028. Thus, we observed significant differences (*p* < 0.05) between the Control and Experimental groups in the initial evaluation (Experimental: 85.86 vs. Control: 93.52) but not in the final evaluation (Experimental: 113.84 vs. Control: 111.88). The Age Group variable was also significant, *F*(2,485) = 149.89, *p* = 0.000, ${\eta }_{p}^{2}$ = 0.382. *Post-hoc* comparisons with the Bonferroni test showed that as student age increased, so did performance in the concentration dimension. Thus, as in the other dimensions analysed, there was a significant improvement (all *p’s* = 0.000) as the participants progressed through the Age Groups (Age Group 1: M = 53.63; Age Group 2: M = 08.25; Age Group 3: M = 152.10). The interaction Group x Age Group was also significant, *F*(2,485) = 3.56, *p* = 0.029, ${\eta }_{p}^{2}$ = 0.1, as well as the interaction Evaluation x Group x Age Group, *F*(2, 485) = 5.46, *p* = 0.05, ${\eta }_{p}^{2}$ = 0.22. As with the other dimensions analysed, it can be seen that in Age Group 1, biofeedback training is more effective than in the other Age Groups. Considering that the Experimental group obtained worse pre-test scores than the Control group, we also observed that Age Group 1 obtained better post-test scores in Concentration than the Control group (Experimental: 76.56 vs. Control: 51.71). Also in Age Group 1, there was a statistically significant post-test improvement in the Concentration measure with respect to the pre-test, and with a large effect size, *t*(66) = −9.44, *p* = 0.000, d = −1.213, higher than in the other Age groups: Age Group 2: *t*(216) = −11.09, *p* = 0.000, d = −0.621; Age Group 3: *t*(44) = −8.07, *p* = 0.000, d = −0.741.

#### Total Test Rate Effectiveness (TOTR)

This measure evaluates the overall effectiveness of the test by subtracting the sum of omissions and errors from the total number of responses. TOTR was higher after than before intervention (120.63 vs. 98.03), *F*(1, 484) = 150.286, *p* = 0.000, ${\eta }_{p}^{2}$ = 0.237. The Group factor was not significant, nevertheless, it interacted with the Assessment factor, *F*(1,484) = 35.51, *p* = 0.000, ${\eta }_{p}^{2}$ = 0.068.

Therefore, in the initial evaluation, the Control group scored better in the TOTR variable than the Experimental group (105.48 vs. 94.01), *t*(552) = −2.927, *p* = 0.004, d = 0.268; however, after training, there were no significant differences between Control and Experimental groups in the final evaluation (117.67 vs. 118.96), *t*(516) = 0.314, *p* = 0.753, d = 0.029. Each group improved their performance in this dimension from the initial to the final assessment, showing a greater impact of training on the Experimental group than on the Control group, Control *t*(159) = −6.69, *p* = 0.000, d = −0.324; Experimental *t*(329) = −16.49, *p* = 0.000, d = −0.588. The Age Group factor was also significant, *F*(2,484) = 130.83, *p* = 0.000, ${\eta }_{p}^{2}$ = 0.351. *Post-hoc* comparisons with the Bonferroni test showed that as the age of the participant increased, the performance in the total effectiveness of the test increased. Thus, students in Age Group 1 performed worse (M = 71.55) than students in Age Group 2 (M = 111.48) and Age Group 3 (M = 154.45). Moreover, students in Age Group 3 also outperformed those in Age Group 2 (all *p’s* = 0.000). Finally, the interaction Group x Age Group x Assessment was significant, *F*(2,484) = 10.07, p = 0.000, ${\eta }_{p}^{2}$ = 0.051. Therefore, in accordance with prior analysis, biofeedback training was more effective in Age Group 1. Given that the Experimental group also obtained worse scores in the total effectiveness of the test, Age Group 1 obtained better post-test results than the Control group (85.32 vs. 68.87), t(113) = 2.86, *p* = 0.006, d = 0.574). Regarding the improvement in total test effectiveness scores of the Experimental group in the post-test compared with the pre-test, Age Group 1 also obtained a very large effect size, *t*(66) = −8.13, *p* = 0.000, d = −1.204), higher than the other Age Groups: Age Group 2: *t*(215) = −12.63, *p* = 0.000, d = −0.631; Age Group 3: *t*(44) = −6.95, *p* = 0.000, d = −0.689).

#### Regression to the Mean

Analysis regarding TN-E, CON, TO, and TOTR might have been biased due to the randomization method employed since, as per school request, some participants within the Experimental group had a baseline TN-E score < 40, which did not occur in the Control group nor in their peers in the Experimental group. Thus, participants with a score < 40 in the Experimental group might have simply performed poorly in the initial assessment and, therefore, their increased performance from the pre- to post-test may not reflect an effect of the intervention but rather a regression to the mean. To account for this, participants in the Experimental group with a score < 40 were excluded from prior analyses (see Table 3) and these were repeated to evaluate the impact of a possible regression to the mean effect. Since repetition of every analysis could result in redundancy, only critical analyses (those that allow us to evaluate the main hypothesis, i.e., Assessment x Group and Three-way) were reported; nevertheless, if inconsistencies with prior analyses were found (i.e., once reliable analysis became unreliable or vice versa) results were reported.

**Table 3:** Means and (standard deviations) of the Experimental and Control groups in the pre- and post-test for the three primary education Age Groups, filtering participants with a TN-E pre-test score < 40

		Experimental			Control		
		Age group 1	Age group 2	Age group 3	Age group 1	Age group 2	Age group 3
		*M(DT)*	*M(DT)*	*M(DT)*	*M(DT)*	*M(DT)*	*M(DT)*
*TN-E*	Pre	69.5(19.1)	102(37)	139(42.5)	73(27.00)	103.14(32.16)	153.85(33.73)
	Post	86.1(26.4)	123(36.8)	168(42.4)	68.87(30.29)	118.92(41.23)	154.35(54.39)
*CON*	Pre	54(24.4)	96.8(40.2)	136(43.6)	42.78(37.28)	95.92(35.04)	148.36(45.41)
	Post	82(28.4)	119(38.3)	165(43.3)	51.72(50.75)	121.09(37.07)	163.67(37.75)
*TO*	Pre	231(23.2)	196(37.4)	160(42.4)	225.22(26.66)	195.86(31.94)	145.09(33.82)
	Post	213(26.6)	165(55)	126(47.9)	229.97(30.31)	152.42(69.10)	116(55.81)
*TOTR*	Pre	69.5(19.1)	102(37)	139(42.5)	73(27.00)	103.14(32.16)	153.85(33.73)
	Post	86.1(26.4)	123(35.9)	168(40.8)	68.87(30.29)	123.59(34.42)	165.78(34.89)

total correctly processed (TN-E), CON concentration (total hits-errors), TO total omissions, TOTR total responses—(omissions + errors)

**Fig. 2:**
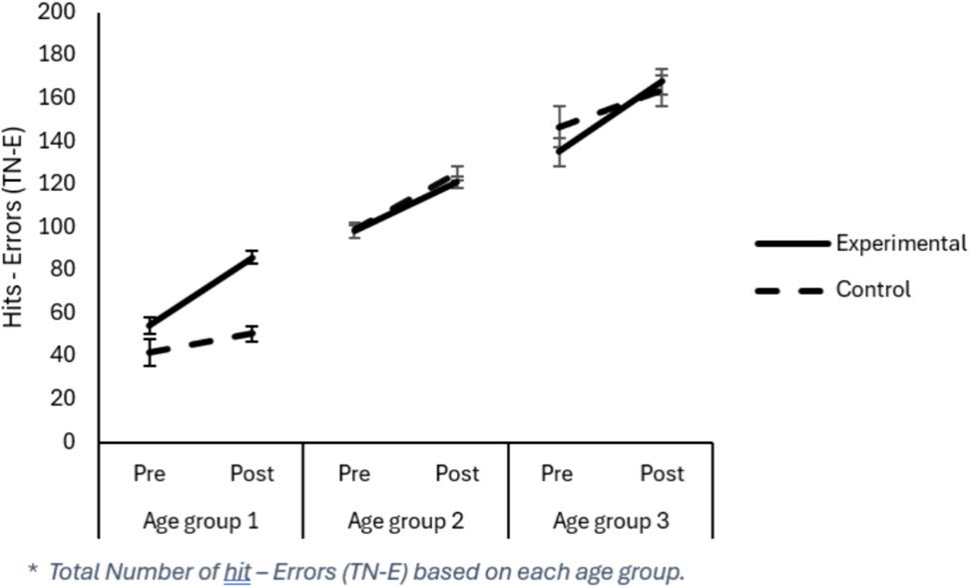
Difference from Pre to Post treatment for the TN-E

**Fig. 3:**
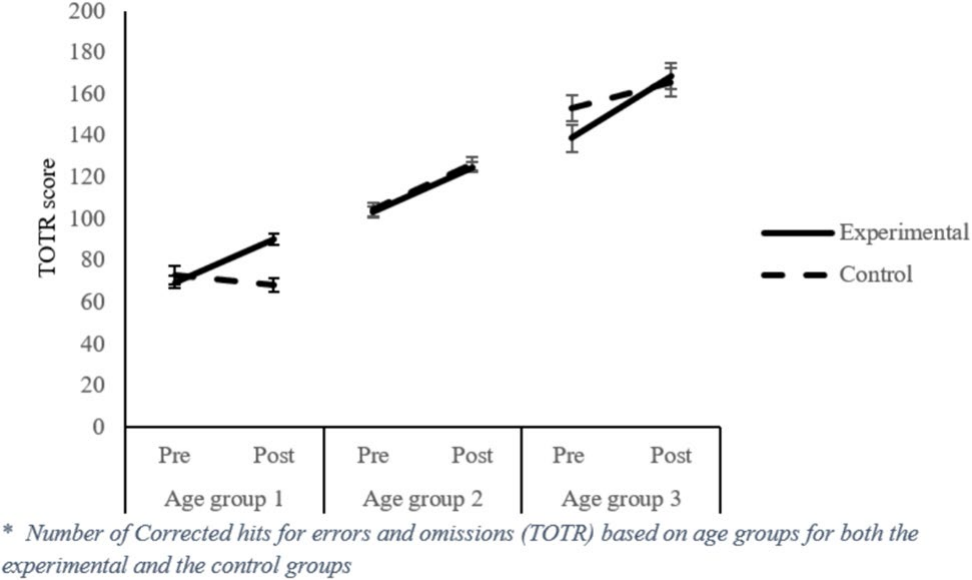
TOTR score per age groups

**Fig. 4:**
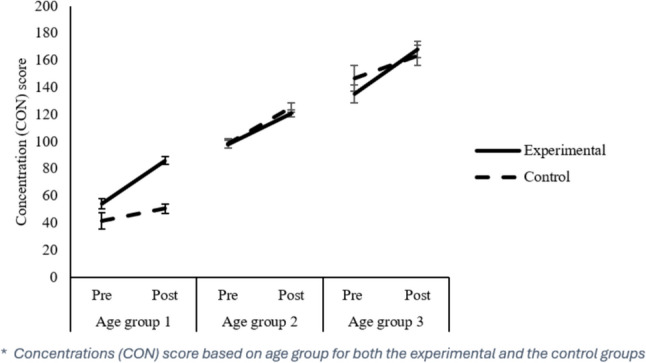
Concentration changes based on age groups

**Fig. 5:**
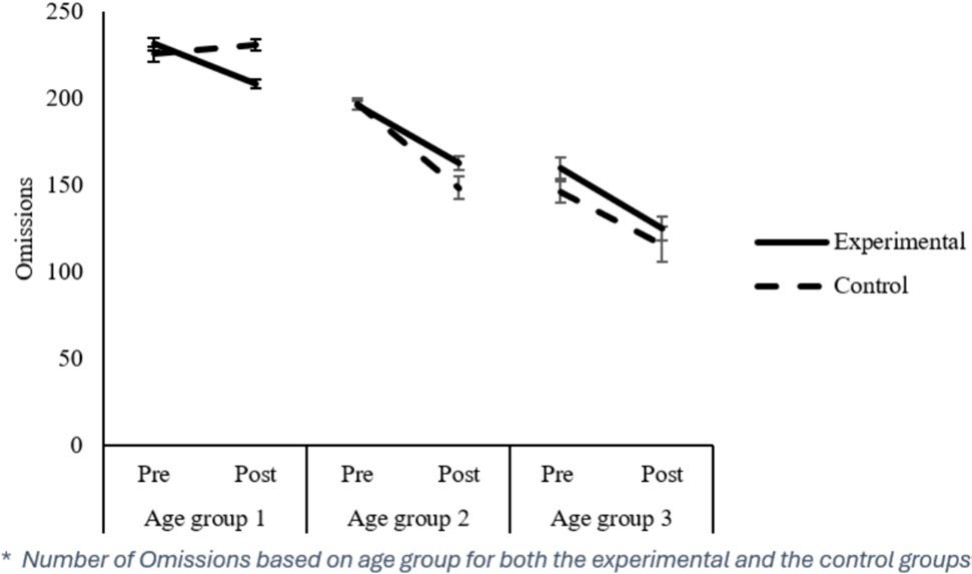
Number of omission for all age group

All critical interactions related to TN-E (see Fig. 2), *F’s* =  ≥ 5.15, *p* ≤ 0.006, ${\eta }_{p}^{2}$ ≥ 0.022, and TOTR (see Fig. 3), *F’s* ≥ 9.62, *p* = 0.000, ${\eta }_{p}^{2}$ ≥ 0.041, remained significant. However, regarding the CON (see Fig. 4) variable, all interactions were reliable, *F’s* ≥ 7.59, *p* ≤ 0.001, ${\eta }_{p}^{2}$ ≥ 0.025, except for the Assessment x Age Group interaction, *F*(2, 448) = 0.703, *p* = 0.496; for the TO (see Fig. 5) variable, the Assessment x Group interaction became unreliable, *F*(1, 483) = 1.05, *p* = 0.307, yet the rest remained significant, *F’s* ≥ 5.44, *p* ≤ 0.005, ${\eta }_{p}^{2}$ ≥ 0.022. Thus, despite no apparent regression to the mean regarding the Total correct answer (TN-E), CON, and TOTR, there might have been a regression to the mean effect.

We previously established that participants in the Experimental group had a poorer performance on the pre-test but, after intervention, were able to perform similarly to their peers. Interestingly, the exclusion of participants with a TN-E test score ≤ 40 revealed that, across all four attention variables (TN-E, TO, CON, and TOTR), groups were differentially impacted based on Age Group, as manifested by the three-way interaction.

**Fig. 6:**
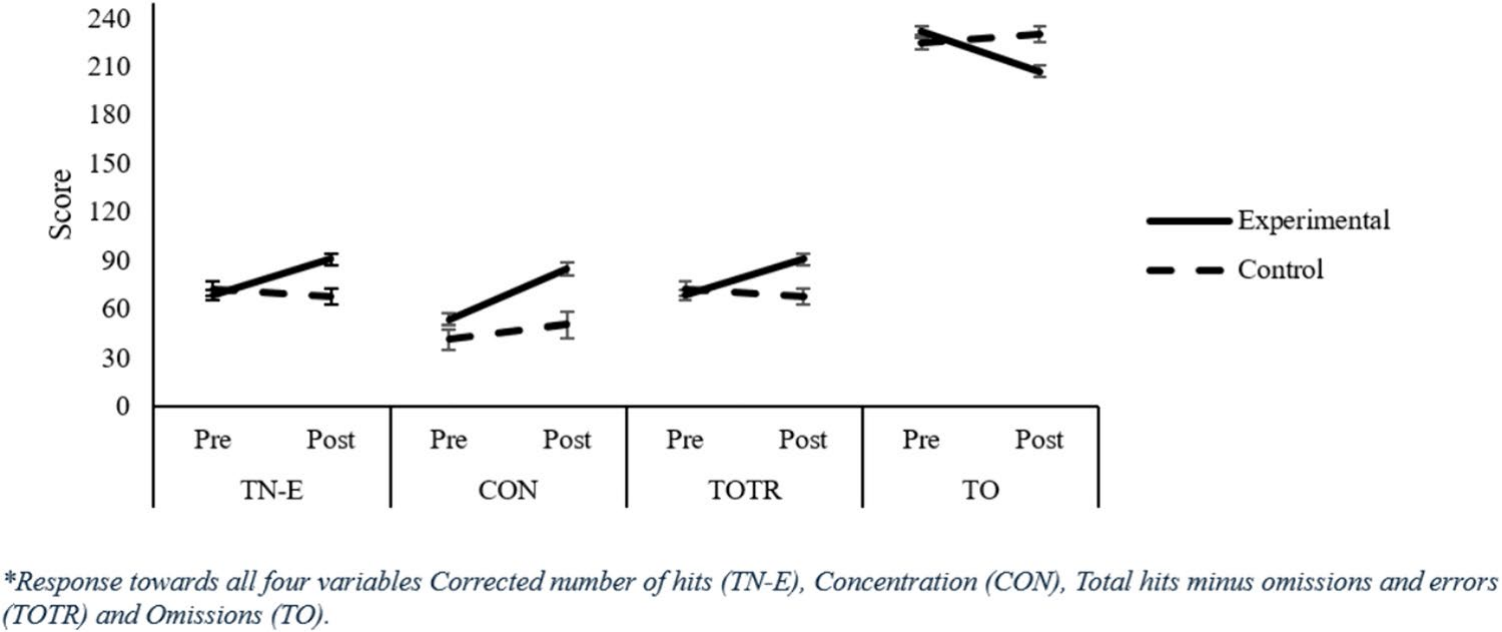
Effectivesness od the intervention for the first cycle for all attention measures

*Post-hoc* analysis revealed that, in the pre-test, Age Group 1 participants (see Fig. 6) scored similarly in number of corrected hits (TN-E; Experimental: 68.98 vs. Control: 73), *t*(93) = −0.85, *p* = 0.40; TOTR (Experimental: 68.98 vs. Control: 73), *t*(93) = −0.85, *p* = 0.40, Omissions (TO; Experimental: 231.70 vs. Control: 225.22), *t*(93) = 1.26, *p* = 0.21, and Concentration (CON; Experimental: 53.67 vs. Control: 42.78) *t*(93) = 1.71, *p* = 0.09. However, after intervention, Experimental Age Group 1 surpassed their counterparts in the Control group for correct hits (TN-E; Experimental: 87 vs. Control: 68.87), *t*(93) = 3.08, *p* = 0.003, d = 0.64, Concentration (CON; Experimental: 76.57 vs. Control: 51.72), *t*(93) = 3.67, *p* = 0.000, d = 0.76, corrected hits minus omissions (TOTR; Experimental: 87 vs. Control: 68.87), *t*(93) = 3.075, *p* = 0.003, d = 0.64, and the number of omissions was reduced (TO; Experimental: 211.47 vs. Control: 229.97), *t*(93) = −3.10, *p* = 0.003, d = −0.64.

To account for how changes in CS impacted d2 measures, new variables were created measuring the increased or decreased score from the pre- to the post-test for both CS (low, medium, and high) and d2 variables (TN-E, TO, TOTR, and CON) and correlation analysis using Pearson’s *r* was applied. This analysis revealed no correlations between the low (*p* ≥ 0.672), medium (*p* ≥ 0.224), or high (*p* ≥ 0.90) CS with any of the d2 measures. Thus, despite the impact that breathing training had on possible attention changes, said relationship was not direct.

Regarding Age Group 2, no significant differences were found between the Experimental and Control groups, neither before (*p* ≥ 0.842) nor after the program (*p* ≥ 0.069).In Age Group 3, there were no differences before (*p* ≥ 0.101) or after intervention (*p* ≥ 0.234).

Finally, as previously mentioned, a particular set of participants, who were requested by the different schools to take part in the experiment and were assigned to the Experimental group, were excluded from the analysis. However, to analyse whether the program was able to benefit these students, additional analyses with this group as the main Experimental group are included. Analysis was performed using a 2 (Group: Control vs. Experimental) × 2 (Assessment: Pre- vs. post-training) mixed ANOVA, and *post-hoc* comparisons were analysed using the Student’s t-test. The Age Group variable was discarded as participants in the Experimental group were all from Age Group 1, therefore, no interesting comparisons could be made. Nevertheless, since prior differential effects of Age Group had been found, the Experimental group was only compared with participants of Age Group 1 in the Control group to equate conditions.

Initial ANOVA analysis revealed significant main effects of Assessment for all variables, *F* ≥ 24.71, *p* = 0.000, ${\eta }_{p}^{2}$ ≥ 0.253, and a main effect of group for TN-E, TO, and TOTR, *F* ≥ 5.85, *p* ≤ 0.018, ${\eta }_{p}^{2}$ ≥ 0.073, but not for concentration (CON), *F*(1, 73) = 1.80, *p* = 0.183. In all cases, there was an interaction (Assessment x Group), *F* ≥ 13.64, *p* = 0.000, ${\eta }_{p}^{2}$ ≥ 0.157.

*Post-hoc* analysis showed that, before training, participants in the Experimental group performed poorly compared with their Control group counterparts in Omissions (TO; Experimental: 273.55 vs. Control: 225.22), *t*(79) = 10.63, *p* = 0.000, d = 2.36, corrected hits (TN-E; Experimental: 25.45 vs. Control: 73), *t*(79) = −10.39, *p* = 0.000, d = −2.31, Total Test Rate Effectiveness (TOTR; Experimental: 25.45 vs. Control: 73), *t*(79) = −10.39, *p* = 0.000, d = 2.31, and Concentration (CON; Experimental: 7.63 vs. Control: 42.78), *t*(79) = −4.36, *p* = 0.000, d = −0.97.

After treatment, participants in the Experimental group significantly reduced their number of omissions (TO; Experimental: 212.26 vs. Control: 229.97), *t*(95) = −2.12, *p* = 0.036, d = −0.44, and performed similarly to their counterparts for the number of corrected hits (TN-E; Experimental: 82.63 vs. Control: 68.87), *t*(94) = 1.96, *p* = 0.053, the total number of corrected hits minus omissions (TOTR; Experimental: 25.45 vs. Control: 73), *t*(94) = 1.96, *p* = 0.053, and Concentration (CON; Experimental: 69.02 vs. Control: 51.72), *t*(79) = 1.97, *p* = 0.052.

Nevertheless, results in this regard must be considered cautiously as comparisons between these two groups could be attributed to an effect of treatment or regression. Moreover, despite the non-significance of groups post-treatment, all comparisons were close to significance.

## Discussion and Conclusions

The HRV biofeedback intervention aimed to improve attention in primary school children. The results of the study show that the intervention developed with HeartMath EnWave software (Institute of HeartMath, 2012) is a simple and effective strategy to modify the way children breathe and influence their HRV across all ages. However, an improvement in attention was only seen in Age Group 1 children. Contrasting with previous studies, which analysed the biofeedback effects based on prolonged and paused breathing in primary education with very small samples (Crevenna et al., 2016) or with non-homogeneous age groups (Rush et al., 2017), this study discriminated the effects between three primary education Age Groups and with a large number of participants.

To understand the results obtained, it is necessary to understand the composition of the Experimental group. The participating school recommended that part of the Experimental group be made up of pupils who, because of their personal situation, needed to improve their attention and academic performance. We considered that, as in previous studies (Lynch & Chen, 2015; Rukmani, et al., 2016; Rush et al., 2017; Wade et al., 2017), the non-randomisation of students in need of attentional improvement and academic performance corresponded to an educational and personal adjustment criterion that we prioritised in this intervention. This acceptance found its empirical foundation in the positive results observed in different biofeedback interventions, like those developed on academic performance and well-being in children with traumatic stress in residential care (Schuurmans, et al., 2020), premature alcohol exposure (Reid & Petrenko, 2018), different types of intellectual disability (Laborde et al., 2017), or a population diagnosed with ADHD (Groeneveld et al., 2019; Lloyd et al., 2010; Price et al., 2017; Rukmani et al., 2016; Wade et al., 2017). Therefore, part of the Experimental group was selected according to the school's criteria. The rest of the Experimental group was selected randomly, as was the Control group.

The inclusion of this group with difficulties might be of interest, as results suggest that they benefited from the program, equating their peers post-treatment—however, remember that these results were close to significance, despite having the power to find differences of 0.16, 0.67, and 0.97 to reveal small, medium and large effects, respectively). Even so, said findings must be considered carefully as results might reflect a regression to the mean effect and not a pure treatment effect. To be able to confidently claim such a conclusion, both Control and Experimental groups should include students with similar conditions.

Upon exclusion of these participants, results showed that, despite successful training in HRV, attention was only improved in children from Age Group 1, not in the others.

An advantage of the design is that it allowed the effect of biofeedback to be studied in all three primary school Age Groups. This distinction made it possible to determine whether the age and developmental stage of the students influenced the effectiveness of the treatment.

Attentional processes are thought to improve with age (Arán Filipetti, 2022; Jiménez et al., 2012; Rivera et al., 2017). For example, Jiménez et al. (2012) tested 1,032 primary school students using the d2 test of attention (Brickenkamp, 2002), finding that performance improved significantly as a function of age. Furthermore, a recent study (Arán Filipetti et al., 2022) provide considerable insight: first, there might be a relationship between TN-E and CON with different measurements of executive function (e.g., D WISC-IV, LNS WISC-IV, and Stroop) and second, high scores in both the TN-E and CON measures are related to increased performance in mathematical problem-solving and reading. Thus, the increased performance in the different components of the d2 test, the results of which suggest that they benefited from treatment, could imply transferable competencies that could impact the academic performance of Age Group 1 students.

The impact on Age Group 1 could be due to developmental factors. To illustrate, children in Age Group 1 (7–8 years) are thought to be developing processes related to their *orientation network* (Posner, 2023), such as inhibitory control. Therefore, our program was able to increase said processes with a physiological approach (HRV) based on the Polyvagal theory and supported by the Model of Neurovisceral Integration. The Biofeedback program, which focuses on the effect that relaxed breathing has on HRV—more specifically the increment of high HRV—allows them to be able to activate the parasympathetic system, thus increasing their ability to regulate parasympathetic activity, enhancing inhibitory control, which in turn has an impact on attention related to the *orientation network* proposed by Posner (2012) and his colleagues (Posner et al., 2020).

Furthermore, the improvement in Experimental group participants, who differed from their peers in the Control group and were included upon school request, makes sense when considering the basis of the present work. As participants in this group had several issues causing a decrement in school performance based on the lack of inhibitory control of several processes (e.g., lack of attention control, lack of emotional control…) the use of a program, which is thought to enhance inhibitory control, should, in theory, be of benefit to them, as suggested by our results.

Concerning the effectiveness of HRV biofeedback interventions for improving attention in the school setting, as mentioned above, only two studies have been conducted with primary school students. Crevenna et al. (2016) conducted a six-week HRV biofeedback programme with 15 fourth-year primary school students aged 10 years, which also aimed to improve attentional capacity as measured by the d2 test (Brickenkamp, 2002). After the intervention, the Experimental group showed significant improvements from baseline to the end of the intervention. Furthermore, this improvement was maintained until the end of the school year, showing that the benefits of the training were maintained over time. The second HRV biofeedback intervention was proposed by Rush et al. (2017) with 27 boys and girls aged 8–12 years. They focused on intervening in students with special needs characterised by poor academic performance and difficulties with social skills. They designed a 12-week training aiming to improve persistence or maintenance in performing a task, as measured by the 'Behavioural Observation of Students in Schools’ (BOOS) (Shapiro, 2011). After the intervention, students in the treatment group spent significantly less time off-task than students in the Control group. However, results in our experiment conclude otherwise, as biofeedback training was only able to impact attentional processes in Age Group 1 but not the rest.

Nevertheless, the previous (Crevena et al., 2016; Rush et al., 2017) and current programmes were not attentional training programs per se but rather influenced those physiological variables thought to impact attention. Thus, attentional training (e.g., Rueda et al., 2012) programmes accompanied by breathing-focused HRV biofeedback training might yield an enhanced improvement in attention.

As far as the limitations of this study are concerned, the main one is the composition of the Experimental group itself; however, exclusion criteria allowed us to somewhat equate group conditions. At the same time, it also confirms the suitability of carrying out HRV biofeedback interventions with students with difficulties. Another limitation of our study stems from one of our main objectives. Considering that critical analyses intended to evaluate the effectiveness of the intervention in a cross-sectional manner, the longitudinal effects of the intervention cannot be evaluated via the present experiment.

Moreover, considering the relevance that age and socioeconomic variables might play (e.g., Arán Filipetti et al., 2022) and given that maternal attachment seems to play a mediating role in HRV (Sichko et al., 2018), it would be of great interest to analyse the influence of maternal-paternal-child relationships on HRV and attentional capacity at this age (6-12 years), as well as that of their age and socioeconomic conditions. It is also worth analysing whether the improvement in attentional capacity has an impact on school performance, school climate, and other realities of the learning process that take place in school. In this way, more knowledge would be gained about the dynamics of biofeedback interventions for HRV, which would allow us to adapt and improve future HRV biofeedback programmes aimed at improving attention in primary education, always based on their effectiveness. Thus, future research should examine the long-term effects of such programmes and the role of other possible mediating variables.

## Data Availability

No datasets were generated or analysed during the current study.
